# Large-bore vacuum-assisted biopsy of axillary lymphadenopathy

**DOI:** 10.1186/bcr2963

**Published:** 2011-11-04

**Authors:** ND Forester, P Burrows, A Jack, A-M Wason

**Affiliations:** 1 Pennine Breast Unit, St Luke's Hospital, Bradford, UK; 2 Haematological Malignancy Diagnostic Services, Leeds, UK

## Introduction

Lymphoma diagnosis conventionally requires nodal excision biopsy, to allow histological subclassification of tumours on samples with preserved tissue architecture. As such, diagnostic accuracy following 16G or 18G core biopsy is difficult. Large-bore vacuum-assisted biopsies, such as Mammotome, can percutaneously sample large volumes of breast tissue with excellent tissue architecture preservation. We describe vacuum-assisted biopsy of axillary nodes to investigate lymphadenopathy in surgically high-risk patients.

## Methods

Eight patients (seven male, one female; median age 52, range 27 to 65) underwent ultrasound-guided, 8G vacuum-assisted biopsy of axillary lymphadenopathy between March 2009 and February 2011. The median largest node size was 28 mm (range 14 to 87 mm). Three patients had previous ultrasound-guided 18G core biopsies, which were insufficient for diagnosis. Between three and 15 cores were obtained (median = 7) and sent fresh to the Haematological Malignancy Diagnostic Services.

## Results

In seven patients, 8G vacuum-assisted biopsy provided sufficient histologically intact nodal material to be fully diagnostic. In one patient, tissue was suspicious for lymphoma, but insufficient for final diagnosis. In seven patients with adequate tissue sampling, six had lymphoma and one had reactive lymphadenopathy. Of the lymphoma diagnoses, four were new diagnoses (two Hodgkin, one follicular, one diffuse large B-cell lymphoma) and two were recurrent lymphomas. No procedure-related complications occurred.

## Conclusion

Ultrasound-guided large-bore vacuum-assisted biopsy can safely biopsy axillary lymphadenopathy. Furthermore, samples obtained have sufficiently preserved tissue architecture to allow a conclusive diagnosis of lymphoma, without requiring surgical intact node excision. In our institution, this technique has proved useful in high-risk surgical candidates, and where nodal size would have made surgery technically difficult.

